# Post‐COVID‐19 Neutropenia in an Infant With Thalassemia Minor: Case Report

**DOI:** 10.1002/ccr3.9668

**Published:** 2025-02-19

**Authors:** Yasmine Elsherif, Omar Elsherif, Mehran Karimi, Ismail A. Ibrahim, Hana J. Abukhadijah

**Affiliations:** ^1^ American Hospital Dubai (AHD) Dubai UAE; ^2^ Tbilisi State Medical University Tbilisi Georgia; ^3^ Faculty of Health Sciences Fenerbahce University Istanbul Turkey; ^4^ Faculty of Health Sciences Lomza State University of Applied Sciences Łomża Poland; ^5^ Medical Research Center Hamad Medical Corporation Doha Qatar

**Keywords:** bone marrow aspiration, coronavirus disease, granulocyte‐colony stimulating factor therapy, neutropenia, post‐viral neutropenia

## Abstract

This case of an 11‐month‐old female who developed severe neutropenia following COVID‐19 infection underscores the need for heightened vigilance and monitoring of hematological parameters in infants post‐COVID‐19. The exact mechanism of COVID‐19‐induced neutropenia is not fully understood, but it may involve cytokine‐induced suppression of hematopoiesis and bone marrow repression due to the inflammatory response. Given the potential for serious clinical implications, including increased susceptibility to infections, it is crucial to effectively identify and manage neutropenia in this vulnerable population. Further research is necessary to elucidate the underlying mechanisms and optimize treatment strategies for COVID‐19‐related hematological complications in infants.

## 1 Introduction

COVID‐19, caused by the SARS‐CoV‐2 virus, emerged in late 2019 in Wuhan, China, and spread rapidly across the globe. Since then, several case reports have highlighted neutropenia as a distinctive feature of COVID‐19 in young infants aged < 1 year. Infants represent a vulnerable population in the COVID‐19 pandemic, with research indicating they comprise a smaller proportion of confirmed cases compared to adults but still pose significant risks [1]. A study involving 1283 infants from the SARSTerPED multicenter database found that during the second wave of the pandemic, hospitalizations of infants increased sixfold. These hospitalized infants were significantly younger on average compared to those in the first wave (3 vs. 8 months, *p* < 0.0001), and they experienced longer hospital stays [2].

Clinical manifestations in infants generally include mild gastrointestinal symptoms, which may be prominent, along with frequently observed skin manifestations. Neurological issues may present with mild signs such as poor feeding, irritability, and/or lethargy [3]. Additionally, neutropenia is noted as a distinct complication of COVID‐19 in infants. Neutrophils play a crucial role in immune defense, and neutropenia occurs when the absolute neutrophil count (ANC) falls below 1500 cells/μL, classified by severity: mild (ANC 1000–1500 cells/μL), moderate (500–1000 cells/μL), severe (< 500 cells/μL), and very severe (< 200 cells/μL) [4], requiring careful management to prevent severe infections. The underlying mechanisms are under investigation, highlighting the complex interplay between viral infections and hematological health. Continued research and vigilant clinical practice are essential to improve outcomes for infants affected by COVID‐19‐related neutropenia.

## 2 Case History/Examination

An 11‐month‐old female was referred to the hematology clinic 1 month post‐SARS‐CoV‐2 infection due to an abnormal complete blood count (CBC) conducted 4 days after her admission to the hospital caused by COVID‐19. She was delivered via C‐section, breastfed for 3 months, and subsequently switched to Neocate due to a cow's milk allergy. She has been receiving iron (Sideral), 10 drops once daily. Her vaccinations are up‐to‐date. The family history includes three older healthy brothers, one with thalassemia minor. The parents are non‐consanguineous and healthy, with a paternal cousin having a thalassemia major. The physical exam was unremarkable; vital signs were stable, with no fever or respiratory distress. She was hydrated, alert, pale in appearance, well matured, and moderately nourished, weighing 7 kg.

Initially, the patient presented to the pediatrician with complaints of cough, runny nose, and high‐grade fever of 40°C, lasting for the past 5 days. She had been in contact with sick individuals, including her mother, who tested positive for COVID‐19, as well as her siblings. She did not appear lethargic upon admission. A polymerase chain reaction (PCR) confirmed COVID‐19. She was treated with intravenous (IV) antibiotics for 2 days and then discharged on oral antibiotic tablets. The mother did not follow up in the clinic afterwards as the symptoms resolved. The mother reported that the COVID‐19 illness course lasted about 10 to 12 days in total.

Three months later, the patient presented with a blanching maculopapular rash on the extremities, arms, and elbows, with a few rashes on the legs. There was no purpura, ecchymosis, or mottled skin, suggesting a viral exanthem or allergy. The rash on the hands and feet lasted for 1 week and was not itchy, with no other concerns or fever. On July 18, 2023, she developed a runny nose, ear pulling, and a low‐grade fever with a maximum temperature of 38.3°C, with no history of cough or rash. On physical examination, her ears showed erythematous and bulging tympanic membranes bilaterally (right more than left), nasal congestion, and normal tonsils with no exudates but positive pharyngitis. She was given oral antibiotic Amox/Clav (AUGMENTIN 457 mg/5 mL) suspension, 2.5 mL orally every 12 h for acute otitis media and was scheduled for a follow‐up within a week. She was seen by an otorhinolaryngology physician, who observed tremendous improvement and complete resolution of the middle ear infection.

Subsequently, she experienced vomiting, non‐bloody diarrhea, and skin rashes followed by persistent fever, poor feeding, and cough, leading to her presentation at the Emergency Department. During this admission, her laboratory results still showed persistent neutropenia, which continued almost 9 months after her COVID‐19 infection. And was again found to be COVID‐19 positive in PCR.

## 3 Methods (Differential Diagnosis, Investigations, and Treatment)

Her CBC revealed leukopenia with neutropenia, with a neutrophil count of 1.9 × 10^9^/L; her C‐reactive protein (CRP) was elevated at 10 mg/L, and procalcitonin levels were mildly elevated.

**FIGURE 1:**
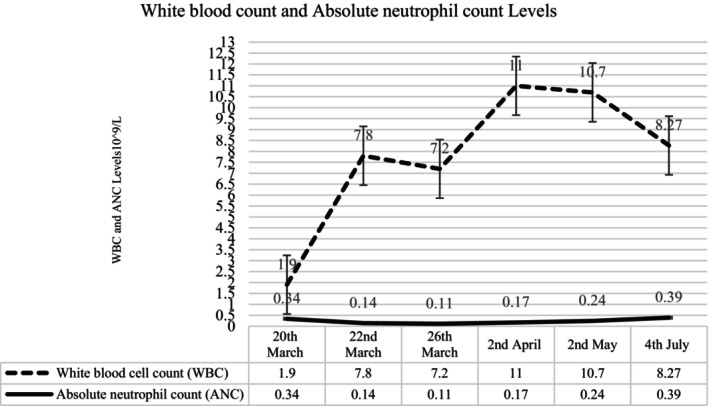
The trend of WBC and A.N.C. during her admission. The neutropenia fluctuated due to an intercurrent viral infection during her follow‐up appointment.

A PCR test on a nasopharyngeal (NP) swab, conducted after disclosing previous contact with COVID‐19 patients, confirmed a diagnosis of COVID‐19 infection, leading to her admission to the pediatric ward. During the hospitalization, incidental findings included severe neutropenia with an absolute neutrophil count (ANC) of 0.14 × 10^9^/L (Figure 1). She was treated with IV ceftriaxone antibiotics for 2 days.

## 4 Results (Outcome and Follow‐Up)

She was discharged the following day on Cefixime (SUPRAX) 20 mg/mL, 70 mg orally for 4 days; ibuprofen (BRUFEN) 20 mg/mL, 70 mg orally three times a day as needed for fever; paracetamol (ADOL) 100 mg/mL oral drops, 100 mg orally every 6 h as needed for 5 days; and sodium chloride nasal solution (Sodium Chloride 0.9% (PHYSIOL) 5 mL inhalation solution) three times a day for 5 days.

Currently, she is post‐COVID‐19 infection but remains neutropenic with an ANC of 0.11 × 10^9^/L and mild anemia, though she is afebrile. She was advised to continue Sideral, 15 drops once daily, and vitamin D. She was also advised to start folic acid, 400 μg once daily, and return for a follow‐up appointment in 1 month to check CBC, hemoglobin electrophoresis, and ferritin.

At her next follow‐up, her ANC slightly improved, with readings of 0.24 × 10^9^/L.

A congenital neutropenia GeneticPanel was performed to evaluate 30 genes associated with severe congenital neutropenia and cyclic neutropenia. The specimen analyzed was white blood cells. The following heterozygous Variants of Uncertain Significance (V.U.S.) were detected:

1. JAGN1 (NM_032492.4), chr3(GRCh37)

0.9935050A>C, c.541A>C, p.Lys181Gln (p.K181Q)

2 LYST (NM_000081.4), chr1(GRCh37)

0.235916419G>T, c.7385C>A, p.Ala2462Glu (p.A2462E)

3 LYST (NM_000081.4), chr1(GRCh37)

0.235872426C>G, c.10108G>C, p.Ala3370Pro (p.A3370P)

Granulocyte antibody results were negative.

**FIGURE 2:**
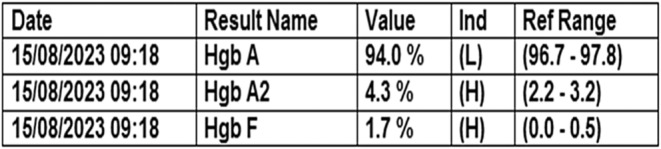
Her hemoglobin electrophoresis is consistent with beta thalassemia minor.

Her hemoglobin electrophoresis is consistent with beta thalassemia minor (Figure 2).

On 31 of December 2023 she received her first dose of Filgrastim (NEUPOGEN) (GCSF) 0.3 mg/0.5 mL PLS, 52.5 mcg = 0.0875 mL, 5 mcg/kg. The following month, on January 28, 2024, she received her second Filgrastim dose (50 μg SC once) without any complications. The mother was instructed to return on February 25, 2024, for the next injection and on March 2, 2024, for a follow‐up and another Filgrastim injection.

**FIGURE 3:**
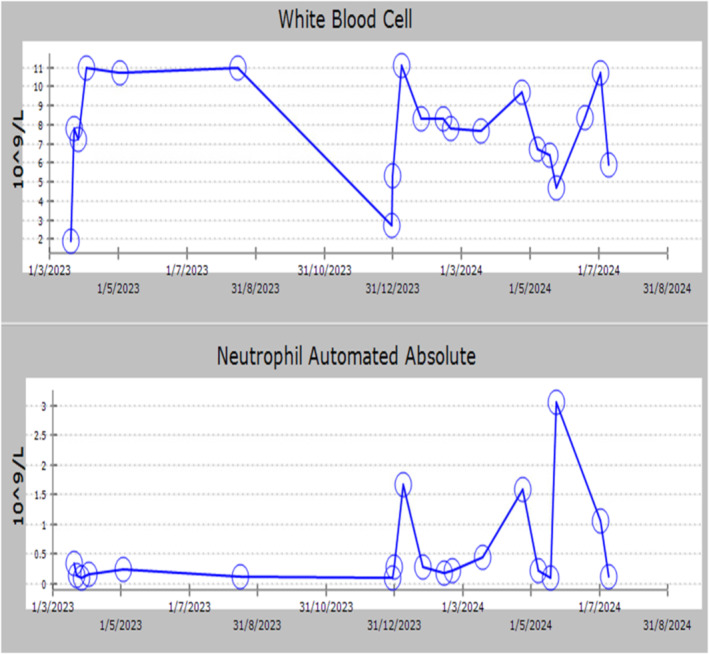
The trend of WBC and A.N.C. during her follow‐up. The neutropenia has been improving after treatment.

The fifth dose was administered on March 12, 2024, followed by another dose on March 18, 2024. From March 18, 2024, and ongoing, she received a dose every 5 days. She received a total of 26 doses of the injection. She responded well to the treatment and was advised to continue it to prevent recurrent infections. The interval between doses was increased to every 10 days (Figure 3). Anti‐neutrophilic antibody and neutropenia genetic tests were negative. However, whole genome sequencing raised suspicion for Chediak‐Higashi syndrome. Bone marrow aspiration smears are cellular with left shifted granulopoiesis and decrease mature neutropeina. There is no evidence of increase blast or abnormal lymphoid population.

Peripheral blood differential: Neutrophils: 10%; lymphocytes: 75%; monocytes: 11%; eosinophils: 3%; basophils: 1%.

Peripheral Smear: Review of peripheral blood smear shows microcytic anemia with anisocytosis. There is adequate leukocyte with absolute neutropenia. No circulating blasts are seen. There is no evidence of abnormal cytoplasmic granules. The lymphocytes are heterogenous including plasmacytoid lymphocytes and large granular lymphocytes: The platelets are adequate with occasional large forms.

Bone marrow aspirate smear differential: Blasts: 1%, promyelocytes: 3%, myelocytes: 20%, metamyelocytes: 11%, bands/neutrophils: 15%, lymphocytes: 14%, monocytes: 2%, eosinophils: 4%, basophils: 0%, erythroid: 30%, and plasma cells: 0%.

Cellularity: Bone marrow aspirate smears are cellular with adequate spicules.

## 5 Discussion

The post‐COVID‐19 sequel in infants remains poorly understood. The youngest case ever reported worldwide was of a 36‐h newborn, while in the United States the youngest case involved a 2‐week‐old infant (8). Henry BM et al. conducted a detailed review of laboratory changes in a pediatric population of SARS‐CoV‐2 patients, reporting a prevalence of neutropenia of approximately 38% among 610 patients reported in 24 studies [5]. Furthermore, Folino, F et al. published a single‐center retrospective study on 93 hospitalized patients aged from 1 month to 18 years with a confirmed diagnosis of SARS‐CoV‐2. This study showed that 12 patients (11 males and 1 female), aged between 38 and 490 days (mean ± SD 160.58 ± 136.51 days), developed neutropenia, estimated at 12.63%. However, both groups cited above developed neutropenia during their initial infection while they were symptomatic, which is a key difference [6].

COVID‐19 is associated with several hematologic abnormalities, with the most commonly reported being lymphopenia (uncommon in infants), thrombocytopenia, and elevated D‐dimer levels [7]. The only hematological abnormality commonly observed in infants is lymphocytosis [8]. Few has been reported about neutropenia post‐COVID‐19, as its mechanism is not yet fully understood, but it is believed that: (1) The virus disrupts the hematopoiesis pathway [9] by directly attacking the stem cells, affecting all the cells at the different lineages, and consequently causing neutropenia and other hematological findings. The expression of ACE2 receptors on diverse cell types, including those in bone marrow, could account for this observation [10]. (2) The immune system may form autoantibodies or complexes of antibodies immune cells that cause the destruction of neutrophils. (3) The sequestration and redistribution of polymorphonuclear neutrophils in extra hematopoietic organs such as the spleen might be one of the mechanism [11].

Differential diagnosis includes the following nutritional deficiencies (vitamin B12 and folate), autoimmune disorders, infections, malignancy, and medication‐induced neutropenia. Each etiology has a different pathophysiology leading to neutropenia. To determine the cause, further investigative tools such as bone marrow biopsy are required to exclude or confirm diagnoses like myelodysplasia, acute leukemias, and marrow failure. The most reliant diagnositic tool is genetic panel.

The c.7385C>A (p.Ala2462Glu) missense variant in the LYST gene has not been reported in affected individuals. The overall minor allele frequency for this variant (rs201821563) is approximately 0.034% with a frequency up to 0.058% in Ashkenazi Jewish sub‐populations (9,10). There is a moderate physicochemical difference between alanine and glutamic acid. This amino acid is moderately conserved across species and an in silico meta‐predictor is inconclusive as to whether this amino acid change impacts protein function.

The c.10108G>C (p.Ala3370Pro) missense variant in the LYST gene has not been reported in affected individuals. The overall minor allele frequency for this variant is approximately 0.003% with a frequency up to 0.01% in Ashkenazi Jewish sub‐populations [12, 13]. There is a small physicochemical difference between alanine and proline. This amino acid is moderately conserved across species and an in silico meta‐predictor is inconclusive as to whether this amino acid change impacts protein function.

The heterozygous c.7385C>A (p.Ala2462Glu) and heterozygous c.10108G>C (p.Ala3370Pro) variants in the LYST gene (MIM:606897) are classified as variants of uncertain significance. The LYST gene encodes lysosomal trafficking regulator, a vesicular transport protein which regulates protein trafficking in lysosomes. Biallelic pathogenic variants in this gene are associated with autosomal recessive Chediak‐Higashi syndrome, which features neutropenia, abnormal bleeding, immune and neurological dysfunction, partial albinism, and giant inclusions within leukocytes that are considered diagnostic for the condition. A severe very early‐onset form is caused by biallelic LYST variants that result in complete loss of protein production or function. Different combinations of biallelic variants that significantly but only partially impair protein function (i.e., “hypomorphic” variants) may cause incomplete forms of the disease that present later in life [14]. Individuals with Chediak‐Higashi syndrome are at high risk for developing hemophagocytic lymphohistiocytosis (HLH). Additionally, there is evidence that some cases [14] of HLH may be attributable to a combination of a single heterozygous pathogenic variant in LYST plus another heterozygous pathogenic variant in another gene involved in the cytotoxic lymphocyte degranulation pathway (PRF1, UNC13D, STX11, STXBP2, AP3B1, or RAB27A) (i.e., digenic inheritance) [15, 16]. This test cannot determine whether the detected variants are in cis (on the same chromosome) or in trans (on different chromosomes). Genetic testing of this individual's parents and/or other first degree relatives could help to clarify this result.

Taken together, the evidence is not sufficient to determine whether these variants are benign or pathogenic. Therefore, these variants are classified as variants of uncertain significance.

Individuals may have a pathogenic variant in one of the interrogated genes that is not detectable by the methods utilized. Additionally, the clinical phenotype that is observed in this individual and/or family may be due to a pathogenic variant or variants in another gene not targeted by this test.

Some case reports have utilized granulocyte‐colony stimulating factor (G‐CSF) therapy to prevent secondary infections. Additionally, prophylactic G‐CSF treatment is sometimes given to prevent unexpected complications in patients having chemotherapy. In our case, a wait‐and‐watch approach is deemed ideal, as neutropenia may resolve without medical intervention in young, healthy individuals, especially since G‐CSF was noted to have fatal complications when administered to post‐COVID‐19 neutropenic patients [17]. Lufti et al. reported the earliest case of agranulocytosis following COVID‐19 infection [18], which was effectively treated with G‐CSF 1 week after symptom resolution [5]. Tocilizumab is thought to help suppress the hyperinflammatory response and reduce mortality, thus recommended for patients with human coronavirus if serum IL‐6 content is over 20 pg/mL despite prolonging neutropenia, even after the resolution of acute symptoms [19]. Lerman et al. demonstrated three cases of connection between the onset of neutropenia and the starting of metamizole therapy [20].

## 6 Conclusion

Recent studies have been emphasizing lymphocytes and clotting factors, with less frequent cases reported of neutropenia in young infants post‐COVID‐19 infection. Thus, reporting these cases is essential for future management, even if they are asymptomatic, due to the risk of secondary severe infections in neutropenic patients.

## Author Contributions

**Yasmine Elsherif:** conceptualization, data curation, resources, writing – original draft, writing – review and editing. **Omar Elsherif:** resources, writing – review and editing. **Mehran Karimi:** conceptualization, data curation, resources, writing – original draft, writing – review and editing. **Ismail A. Ibrahim:** resources, writing – original draft, writing – review and editing. **Hana J. Abukhadijah:** writing – review and editing.

## Ethics Statement

The authors have nothing to report.

## Consent

Written consent form was obtained from the parents and can be provided upon request.

## Conflicts of Interest

The authors declare no conflicts of interest.

## Data Availability

Data available on request from the authors.

## References

[1] M. U. Bhuiyan , E. Stiboy , M. Z. Hassan , et al., “Epidemiology of COVID‐19 Infection in Young Children Under Five Years: A Systematic Review and Meta‐Analysis,” Vaccine 39, no. 4 (2021): 667–677. 33342635 10.1016/j.vaccine.2020.11.078 PMC7833125

[2] M. Sobolewska‐Pilarczyk , M. Pokorska‐Śpiewak , A. Stachowiak , et al., “COVID‐19 Infections in Infants,” Scientific Reports 12, no. 1 (2022): 7765. 35546159 10.1038/s41598-022-11068-0 PMC9094122

[3] N. Cimolai , “COVID‐19 Among Infants: Key Clinical Features and Remaining Controversies,” Clinical and Experimental Pediatrics 67, no. 1 (2024): 1–16. 38013408 10.3345/cep.2023.00794 PMC10764668

[4] N. Singh , S. Singh Lubana , and L. Dabrowski , “Isolated Chronic and Transient Neutropenia,” Cureus 11, no. 9 (2019): e5616. 31720132 10.7759/cureus.5616 PMC6823038

[5] B. M. Henry , S. W. Benoit , M. H. S. de Oliveira , et al., “Laboratory Abnormalities in Children With Mild and Severe Coronavirus Disease 2019 (COVID‐19): A Pooled Analysis and Review,” Clinical Biochemistry 81 (2020): 1–8. 32473151 10.1016/j.clinbiochem.2020.05.012 PMC7251358

[6] F. Folino , C. Menis , G. M. di Pietro , R. Pinzani , P. Marchisio , and S. Bosis , “Incidental Occurrence of Neutropenia in Children Hospitalised for COVID‐19,” Italian Journal of Pediatrics 48, no. 1 (2022): 43. 35292084 10.1186/s13052-022-01234-5 PMC8922397

[7] M. Yang , C. Li , K. Li , et al., “Hematological Findings in SARS Patients and Possible Mechanisms (Review),” International Journal of Molecular Medicine 14, no. 2 (2004): 311–315. 15254784

[8] C. Kosmeri , E. Koumpis , S. Tsabouri , E. Siomou , and A. Makis , “Hematological Manifestations of SARS‐CoV‐2 in Children,” Pediatric Blood & Cancer 67, no. 12 (2020): e28745. 33009893 10.1002/pbc.28745 PMC7646039

[9] L. H. A. Cavalcante‐Silva , D. C. M. Carvalho , É. A. Lima , et al., “Neutrophils and COVID‐19: The Road So Far,” International Immunopharmacology 90 (2021): 107233. 33290963 10.1016/j.intimp.2020.107233 PMC7703515

[10] A. P. Yang , J. P. Liu , W. Q. Tao , and H. M. Li , “The Diagnostic and Predictive Role of NLR, d‐NLR and PLR in COVID‐19 Patients,” International Immunopharmacology 84 (2020): 106504. 32304994 10.1016/j.intimp.2020.106504 PMC7152924

[11] C. Huang , Y. Wang , X. Li , et al., “Clinical Features of Patients Infected With 2019 Novel Coronavirus in Wuhan, China,” Lancet 395, no. 10223 (2020): 497–506. 31986264 10.1016/S0140-6736(20)30183-5 PMC7159299

[12] S. T. Sherry , M. Ward , and K. Sirotkin , “dbSNP‐Database for Single Nucleotide Polymorphisms and Other Classes of Minor Genetic Variation,” Genome Research 9, no. 8 (1999): 677–679. 10447503

[13] K. J. Karczewski , L. C. Francioli , G. Tiao , et al., “The Mutational Constraint Spectrum Quantified From Variation in 141,456 Humans,” Nature 581, no. 7809 (2020): 434–443. 32461654 10.1038/s41586-020-2308-7 PMC7334197

[14] W. J. Introne , W. Westbroek , C. A. Groden , et al., “Neurologic Involvement in Patients With Atypical Chediak‐Higashi Disease,” Neurology 88, no. 7 (2017): e57–e65. 28193763 10.1212/WNL.0000000000003622 PMC5584077

[15] K. Zhang , S. Chandrakasan , H. Chapman , et al., “Synergistic Defects of Different Molecules in the Cytotoxic Pathway Lead to Clinical Familial Hemophagocytic Lymphohistiocytosis,” Blood 124, no. 8 (2014): 1331–1334. 24916509 10.1182/blood-2014-05-573105 PMC4141517

[16] E. A. Steen , M. L. Hermiston , K. E. Nichols , and L. K. Meyer , “Digenic Inheritance: Evidence and Gaps in Hemophagocytic Lymphohistiocytosis,” Frontiers in Immunology 12 (2021): 777851. 34868048 10.3389/fimmu.2021.777851 PMC8635482

[17] H. M. Lazarus and R. P. Gale , “G‐CSF and GM‐CSF Are Different. Which One Is Better for COVID‐19?,” Acta Haematologica 144, no. 4 (2020): 355–359. 32791509 10.1159/000510352 PMC7490498

[18] F. Lutfi , A. Benyounes , N. Farrukh , J. Bork , and V. H. Duong , “Agranulocytosis Following COVID‐19 Recovery,” Cureus 12, no. 7 (2020): e9463. 32874794 10.7759/cureus.9463 PMC7455389

[19] B. Atallah , W. el Nekidy , S. I. Mallah , et al., “Thrombotic Events Following Tocilizumab Therapy in Critically Ill COVID‐19 Patients: A Façade for Prognostic Markers,” Thrombosis Journal 18 (2020): 22. 32922212 10.1186/s12959-020-00236-9 PMC7479301

[20] T. T. Lerman , M. Sagi , Y. Shafir , et al., “A Possible Increased Risk of Metamizole‐Associated Neutropenia Among COVID‐19 Patients,” British Journal of Clinical Pharmacology 87, no. 7 (2021): 2902–2906. 33332642 10.1111/bcp.14703

